# Pre-service EFL teachers’ emotional experiences, emotion regulation strategies and influencing factors in research: A Chinese case study

**DOI:** 10.1016/j.heliyon.2024.e36843

**Published:** 2024-08-26

**Authors:** Renquan Heng, Qi Cheng, Liping Pu, Qingyu Tang

**Affiliations:** a School of Foreign Languages, Soochow University, Suzhou, 215006, China; b Faculty of Education, Beijing Normal University, Beijing, 100875, China; c Soochow College, Soochow University, Suzhou, 215006, China; d Suzhou Industrial Park Xinghai Experimental Senior High School, Suzhou, 215124, China

**Keywords:** Emotional experience, Emotion regulation, Research, Pre-service EFL teachers

## Abstract

Currently, teacher emotion in the context of conducting research has drawn much attention in the field of teacher development. However, existing empirical studies mainly focus on in-service teachers’ emotional experiences in conducting research, thus overlooking pre-service teachers. This study involving seven pre-service English as a foreign language (EFL) teachers from a comprehensive university in mainland China as participants adopts a qualitative method to explore their emotional experiences, emotion regulation strategies and influencing factors in research. The first major finding is that the participants experienced positive, negative and complex emotions throughout their research journeys. The negative emotions left the deepest impression, with confusion being the most common emotion. Second, the participants employed both antecedent-focused and response-focused strategies to regulate their negative emotions and sustain the positive ones, but they favored the response-focused strategies. The antecedent-focused strategies included attention deployment, cognitive change, separation, expectation lowering and situation selection, while response-focused strategies cover communication, self-improvement, action taking, relaxation, adaptation and expressive suppression. Third, both personal and environmental factors played a part in the participants’ emotion regulation in conducting research. Personal factors included participants’ past experiences, agency, and personality, while environmental factors included relationships with others and the research support they received. The findings have some implications which may be helpful for pre-service EFL teachers, educators and policy makers in conducting and supervising research.

## 1 Introduction

As positive psychology advances quickly, researchers are giving increasing attention to teachers’ emotional development [1]. In other words, teacher emotion has attracted widespread concern. Teaching is a profession demanding emotional labor [2,3], and the same holds good for doing research. As a crucial concern in the professional development of English as a foreign language (EFL) teachers, carrying out research may evoke various emotions, both positive and negative ones [4]. In the Chinese context, EFL teachers seem to perform more emotional labor than teachers of other subjects, such as mathematics and physics, as English is a foreign language for both the teachers and students. Considering that different emotions may influence EFL teachers’ personal well-being and research, there is an urgent need for teachers to regulate their emotions appropriately.

To date, quite a few discoveries have been made concerning emotion regulation in teaching. These mainly fall into three categories: the effects of teacher emotion regulation [5,6], the goals of teacher emotion regulation [[7], [8], [9]], and teacher emotion regulation strategies [[10], [11], [12]]. However, most studies focus on in-service teachers, with few exploring pre-service teachers’ emotional experiences. As teachers at different stages of their professional development experience different emotions [13], it is of crucial significance to conduct research on teachers at other stages of development, especially those at the pre-service teaching stage. Cultivating pre-service teachers as researchers is crucial because it directly influences their research capabilities, career development and even the quality of basic English education [14]. They function as a special group in the construction of teaching staff, with their development having a direct bearing on that of in-service teachers. Besides, the vast majority of studies concerning teacher emotion probe into the emotional experiences in teaching rather than in research. Furthermore, the existing studies on teacher research mostly focus on research cognition and research practice, giving scant attention to research emotion [15,16]. Since different research emotions can exert varied significant influences on the quality of teachers’ research and education, there is a dire need to examine teachers’ research emotions is in dire need.

Therefore, this qualitative study aims to explore Chinese pre-service EFL teachers’ emotional experiences and emotion regulation strategies in conducting research. The hope, from a theoretical perspective, is that the findings can provide researchers interested in teacher emotion regulation with a better understanding of this topic. Practically, this work might have some implications for pre-service EFL teachers, educators, and policymakers to enhance their work. Specifically, the study is intended to answer the following questions:

(1) What emotional experiences do pre-service EFL teachers have in conducting research?
(2) What emotion regulation strategies do pre-service EFL teachers adopt in research?
(3) What are the possible factors influencing pre-service EFL teachers’ emotion regulation in research?

## 2 Literature review

### 2.1 Teacher emotion and teacher research emotion

Scholars have conceived diverse conceptualizations of teacher emotion from different perspectives. From the perspective of individual psychology, teacher emotion is confined to teachers’ internal psychological experiences, which mainly comprise five components: appraisal, subjective experiences, physiological changes, expressions of emotion and action tendencies [17]. This understanding, however, fails to account for the interaction between teacher emotion and external sociocultural contexts. From the sociocultural perspective, teacher emotion is seen not simply as driven by physiological instinct or impulse but mainly as hinging on sociocultural situations [13]. This is an improvement because it places teachers in a world of social interaction to understand their expressions of emotion, but it unfortunately also deems teacher emotion a direct product of society, ignoring the teachers’ agency. In a post-structuralist view, teacher emotion is a kind of discursive practice in which teachers interact with society [18]. This process is also constrained by some emotional rules and shaped by the interplay of politics, culture and other power relations within the broader context [19]. From an ecological dynamic system perspective, the internal mechanism for the production of teacher emotion lies in its interaction and transaction with the environment [20]. For a complicated system comprising numerous relationships, the environment can be divided into five subsystems: the microsystem, mesosystem, exosystem, macrosystem, and chronosystem [21,22]. This analysis sheds light on the roles that different layers of the ecological environment play in constructing teacher emotion. In light of the third and fourth conceptualizations, teacher emotion in this study is defined as the individual experience of the teacher interacting with the complex environment surrounding them and having the potential to make transformations in their learning, teaching, and researching practice.

Based on the aforementioned conceptualizations of teacher emotion, teacher research emotion in this study mainly refers to teachers’ subjective experiences in their research life. It not only comprises the dynamic interaction between teachers and their complex environment but also involves their capacity to navigate different emotional experiences and become better teachers.

### 2.2 Related studies on teachers’ emotional experiences in research

Teacher emotion, in most cases, is demonstrably either positive or negative [23], though some researchers consider it a continuum rather than a seesaw. Gao et al. [24] conducted a systematic investigation into Chinese EFL teachers’ perceptions of research by conducting in-depth interviews with 28 university EFL instructors. They found that doing research can elicit some negative emotions in teachers, such as confusion and helplessness. Similarly, Li’s [25] qualitative study revealed that Chinese EFL instructors at private universities often experienced negative emotions in their fledgling stage of doing research, with teaching and other miscellaneous work plunging them into a confusing state of not knowing how to strike a balance between teaching and doing research. By contrast, some research has shown that doing research can trigger positive and complex emotions. Gu [26] conducted a qualitative study investigating the emotional experiences of 12 Chinese college instructors pursuing research, and the participants experienced not only negative emotions, like anxiety and frustration, but also positive and complex ones.

However, the participants in previous studies were all in-service teachers, leaving pre-service teachers overlooked. Considering the lack of a systematic and comprehensive learning of how to do research, pre-service EFL teachers may have different or even more complex emotions than in-service teachers, which is the focus of the present study.

### 2.3 Related studies on teachers’ emotion regulation strategies

Since little is known about the emotion regulation strategies teachers adopt in research, this section reviews some related studies on teachers’ emotion regulation strategies in instruction. These studies mostly follow the framework of Gross [27,28], where the regulation process falls into two main categories: one that is antecedent-focused, which means regulating emotions prior to the activation of certain emotional responses, and one that is response-focused, which means regulating emotions after the occurrence of certain emotional responses. Antecedent-focused emotion regulation can be further divided into four points: situation selection, situation modification, attention deployment and cognitive change. Response-focused emotion regulation is also called response modulation, with expressive suppression being the most typical form.

Sutton [7] conducted semi-structured interviews with 30 middle school teachers in the United States to determine their emotion regulation strategies and found that the teachers mainly used four emotion regulation strategies in class – namely, situation modification, attention deployment, cognitive change and response modulation – among which situation modification was the most effective strategy. Similarly, in Finland, Jiang et al. [10] employed surveys and interviews to investigate four teachers’ emotion regulation strategies from the perspectives of both the teachers and their students. According to their results, the teachers mainly used five emotion regulation strategies: situation selection, situation modification, attention deployment, cognitive change and suppression. They also found that antecedent-focused strategies were more effective than response-focused ones, with cognitive change being more effective than suppression.

Taxer and Gross [9] conducted a study on teachers’ emotion regulation strategies through interviews with 56 elementary and secondary school teachers in western and southern regions of the United States. The teachers reported using five emotion regulation strategies, which were similar to those uncovered by Jiang et al. [10]. However, the most frequently used strategy was suppression, which may be detrimental to teachers’ well-being [29,30]. Likewise, Gong et al. [8] performed semi-structured interviews with 34 teachers in elementary, middle and high schools to assess their emotion regulation strategies. The most commonly used strategies were situation selection, situation modification, attention deployment, cognitive change and suppression. However, they used suppression – that is, holding back negative emotions – most frequently, supporting the findings of Taxer and Gross [9]. In the same vein, Uzuntiryaki-Kondakçı et al. [31] utilized a multiple-case design to investigate three in-service teachers’ emotion regulation strategies in their instruction in Turkey, with data collected through diaries, video recordings, field notes and interviews. Their results indicated that the teachers mainly employed five emotion regulation strategies: situation selection, situation modification, attention deployment, cognitive change and response modulation.

In sum, previous studies concur that emotion regulation strategies in instruction mainly fall into two broad categories – antecedent-focused and response-focused – although the subcategories differ slightly from study to study. The most frequently used strategies reported by teachers from different countries are rather diverse. Therefore, it is worthwhile to explore whether cultural background plays a role in the choice of emotion regulation strategy in the Chinese context.

### 2.4 Related studies on the influencing factors of teacher emotion regulation

A close examination of existing studies on the influencing factors of teacher emotion regulation suggests that two factors are at play: personal and environmental factors [3,26,29,32]. Regarding personal factors, teachers’ agency is considered to be an influencing factor. Gu [26] carried out a case study to examine the emotion regulation strategies used by 12 university EFL instructors in eastern China. The analysis of the participants’ interview transcripts indicated that emotion regulation in research was closely related to the participants’ agency. Additionally, teachers’ past experiences may influence emotion regulation. Hosotani and Imai-Matsumura [32] conducted a qualitative study to explore the emotion regulation experiences of 24 high-quality Japanese elementary school teachers in instruction. Their results indicated that the teachers’ previous teaching experiences had quite an impact on their understanding and practice of emotion regulation.

Apart from personal factors, environmental factors seem to be at play in teachers’ emotion regulation. Chang’s [29] quantitative study on 412 Chinese secondary school teachers’ emotional experiences in Hong Kong revealed that the participants’ intimate relationships with their colleagues could influence their emotion regulation. Research policy has also been identified as a contributor to teachers’ emotion regulation. For example, Gu [26] pointed out that the school’s research policy could accelerate the generation of some teachers’ negative emotions.

In light of the aforementioned studies, the authors developed the following conceptual framework (see Table 1) to guide the data collection and analysis in the present study.

**Table 1:** Conceptual framework.

Domains	Analytical Categories	Subcategories
Emotional experiences	Positive emotions	Happiness, motivation …
	Negative emotions	Anxiety, frustration …
	Complex emotions	Pain and happiness …
Emotion regulation strategies	Antecedent-focused	Situation selection
		Situation modification
		Attention deployment
		Cognitive change
		Expectation lowering
		Separation
	Response-focused	Suppression
		Adaptation
		Action taking
		Communication
		Relaxation
Influencing factors	Personal	Agency
		Past experiences
	Environmental	Relationships with others
		Research support
		Expressive rules

## 3 Methodology

### 3.1 Setting and participants

This study was set in the School of Foreign Languages of a key university in mainland China when the participants selected were in the process of doing their various research projects, including a College Students’ Innovative Entrepreneurial Training Plan Programme (Project One), College Students’ Extracurricular Academic Research Fund Programme (Project Two) and an undergraduate thesis (Project Three). According to the principle of purposive sampling [33], which advocates for choosing representative samples that can offer the most relevant information in light of the research purposes, seven pre-service EFL teachers were selected to complete narrative questionnaires and participate in semi-structured interviews and reflections. The participants were all majoring in English education and were pursuing becoming English teachers after graduation. In addition, according to the narrative questionnaires they filled in, they had relatively rich experiences of doing research and ample emotional stories to share, suggesting that they would be able to provide as much meaningful and useful information as possible. Each participant was required to sign a written informed consent form and was informed about the aim of the study and their right to withdraw their participation at any time. The participants’ basic information is shown in Table 2.

**Table 2:** Basic information about the participants.

Participant	Gender	Age	Research Time (Month)	Research Project
Emma	Female	21	10	Project Three
Missy	Female	21	15	Projects Two, Three
Ossy	Female	22	10	Project Three
Tyra	Female	21	15	Projects Two, Three
Iris	Female	22	20	Projects One, Three
Olivia	Female	22	12	Projects One, Three
Neal	Male	22	9	Project Three

### 3.2 Data collection and data analysis

In this study, three kinds of instruments were used: a narrative questionnaire, semi-structured interview and reflection. The narrative questionnaire was modeled on that of Gu et al. [34] with several adjustments made in consideration of the participants’ being pre-service teachers, as shown in the Appendix.

The semi-structured interview questions were designed based on the analytical categories and subcategories used by Gross [27,28] and Gu [26], as shown in the Appendix.

Concerning the reflections, the researchers asked the participants to write at their leisure at least two reflections with no word limit. They were encouraged to write about what happened during their research journey, what their feelings were about these stories, how they managed to navigate their emotional problems, why they chose to regulate in this way and so on. The basic information about the data collection appears in Table 3.

**Table 3:** Basic information about the data collection.

Participant	Narrative Questionnaire	Semi-structured Interview		Reflection	
	Words	Length (Minutes)	Words	Number	Words
Emma	1,844	43	10,106	2	1,244
Missy	1,826	40	8,549	2	1,359
Ossy	1,225	35	7,697	2	775
Tyra	1,087	45	10,736	2	2,011
Iris	2,060	40	11,411	2	1,560
Olivia	1,297	60	11,858	2	1,513
Neal	1,830	32	7,655	3	1,320
Total	11,169	295	68,012	15	9,782

The data analysis was conducted at the same time as the data collection, which was an ongoing process. Data collected through the semi-structured interviews were coded with the help of NVivo 12 software, with the narrative questionnaires and reflections reviewed for triangulation. Below is a brief explanation of the coding process used for the interview transcripts.

The first step was called open coding. The authors carefully read the transcripts with an open attitude and tried to extract quotes directly from them [32], arriving at 23, 11 and 5 first-level codes respectively relating to the three research questions. For example, Missy mentioned in the interview that “At the very start of my research experience, I was definitely imbued with curiosity and passion because the topic of my research project had not been decided yet. I was at a stage where I was curious about everything and wanted to do research on everything”. In this case, “curiosity” and “passion” were directly extracted from the transcript. The second step was called axial coding. The authors compared and analyzed the first-level codes, established connections between these concepts and determined their general categories [32]. Here, “curiosity” and “passion” fell into the category of “positive emotions”. The third step was called selective coding. The authors further analyzed the relationships between these conceptual categories to identify a more abstract and explanatory one [32]. Positive emotions were classified as “emotional experiences”.

## 4 Results

### 4.1 Emotional experiences of pre-service EFL teachers in conducting research

According to the data analysis, all seven pre-service EFL teachers experienced positive, negative and complex emotions in their research (see Table 4), among which negative emotions were mentioned the most – that is, 103 times. These negative emotions included confusion, anxiety, frustration, pressure, boredom, self-doubt, annoyance, helplessness, panic, tiredness, pain and collapse. The second most frequently mentioned emotional experiences were positive, including curiosity, a sense of achievement, motivation, passion, expectation, happiness, satisfaction, pride and surprise. The third type comprised complex emotional experiences: one related to pain and happiness, the other concerning love and hate.

**Table 4:** Coding results of teachers’ emotional experiences in research.

Emotional Experiences (194)		Coding Source	Coding Number	Percentage
Negative (103)	Confusion	7	22	21.36
	Anxiety	6	20	19.42
	Frustration	6	17	16.50
	Pressure	4	8	7.77
	Boredom	3	6	5.83
	Self-doubt	3	6	5.83
	Annoyance	3	5	4.85
	Helplessness	3	5	4.85
	Panic	3	5	4.85
	Tiredness	3	4	3.88
	Pain	2	3	2.91
	Collapse	2	2	1.94
Positive (79)	Curiosity	6	14	17.72
	A sense of achievement	6	13	16.46
	Motivation	5	12	15.19
	Passion	5	10	12.66
	Expectation	6	8	10.13
	Happiness	4	8	10.13
	Satisfaction	4	6	7.59
	Pride	2	4	5.06
	Surprise	2	4	5.06
Complex (12)	Pain and happiness	4	7	58.33
	Love and hate	4	5	41.67

#### 4.1.1 Negative emotional experiences

The coding results indicate that the participants universally experienced negative emotions in doing research, and these were much more profound than their experiences of positive and complex emotions. In addition, these negative emotions were strongly felt during the fledgling and progressing stages of research, when the participants encountered problems with topic selection, literature reading, data collection and data analysis. Altogether, 12 different negative emotions were retrieved from the data. The top three, which the participants mentioned the most often, are subsequently reported and analyzed.

##### 4.1.1.1 Confusion

The seven pre-service EFL teachers mentioned experiencing confusion during the research journey 22 times. Confusion, to a certain extent, originates from a lack of systematic learning of how to conduct research, which manifests in the fledgling and progressing stages. These novices reported that when they had to select a research topic, they felt a strong sense of confusion because they had no idea which research field or research topic to focus on. Tyra shared her emotional experience of confusion about topic selection:

When we were required to submit our selected topic, I only had a slight understanding of research. What I knew was that I could choose a topic related to linguistics, literature or translation. At that time, I found myself interested in pedagogy – namely, to investigate teaching and learning – but I was at a loss about which specific area to narrow down to. This made me imbued with confusion.

Although the participants found a topic which fascinated them, both internal and external factors could precipitate the feeling of confusion. For example, Neal’s array of inner questions can be deemed as a demonstration of his puzzlement:

First of all, I had no criteria upon which to judge whether I was doing research up to standard, which made me further question whether what I was doing currently was meaningful or not. Secondly, without definite goals, I was ill-equipped to carry out my research project. All these obstacles intensified my confusion to a great extent.

Moreover, doubt from instructors could exacerbate this confusion. As Missy mentioned, the feedback offered by her project instructor dampened her enthusiasm somewhat:

After determining a topic, I went to my instructor for his opinions. However, he raised an objection to the feasibility of my design, pointing out that I was not equipped with the capacity to undertake such a demanding project at that time. This, to be honest, made me very confused about my future research area. My original thought was to choose a topic that probably most people had not dabbled in before, but due to the limited access I had, I had no option but to abandon this idea.

In the process of doing research, reading literature and collecting data and analyzing data are likely to evoke some negative emotions. For instance, when Olivia read literature papers and found that the mainstream trend did not run parallel to her study, she was plunged into deep reflection:

I focused on the study of feminist literature, but from the perspective of some well-established scholars, this kind of research appears to be in rebellion against the traditional ideas. Some regarded feminist literature as antagonistic, which made me reflect on the significance of my study. I began to wonder whether I had the determination and perseverance to dig deep into the essential things, in which case I was thrown into a sense of confusion.

Regarding data analysis, many participants expressed their deep confusion about what tools they should turn to for help and the procedures for processing data. Emma, for example, discussed her confusion about how to deal with the collected audio data:

In my study, I need to analyze the audio files recorded by my subjects. For example, I have to conduct data coding according to the accuracy, fluency and complexity of their monologues, which cannot be measured simply via NVivo. Therefore, I have a feeling of being lost in a fog and am totally ignorant of how to do data analysis. Worse still, the sense of confusion weakens my confidence in successfully finishing this project.

As the participants’ descriptions vividly show, doing research inevitably brought these pre-service EFL teachers a feeling of confusion, a very common emotion especially for research novices. This may be partly due to their lack of research learning and experience.

##### 4.1.1.2 Anxiety

Apart from confusion, pre-service EFL teachers were struck with a feeling of anxiety, which was noticeable to them during the progressing stage of research. Six participants referred to this type of emotional experience 20 times, especially when they were reading literature and conducting data collection and analysis. In the case of reading literature, the difficulty of finding papers and reading papers of high quality and with complicated technical terms added to their sense of anxiety. For Neal, reading literature was an anxiety-inducing task:

For me, reading papers turned out to be a boring thing, especially when it came to some topics that I myself was not interested in. Piles of data and the seemingly advanced and complicated English words in these papers flooded me with anxiety.

The data collection process required the participants to interact with others, and many unexpected things would occur. Missy mentioned in the interview one of her emotional experiences of being full of anxiety, which mainly stemmed from external pressure:

During the mid-term assessment, I needed to submit a report on the progress of our project. If my memory serves me right, I had already designed the questionnaires and was about to hand them out at that time. But when I learned that some teams’ data collection work was nearly finished and ours had not started yet, anxiety welled up in my heart.

Data analysis, especially for those conducting a quantitative study, seemed like a troublesome job, which Missy experienced when she started to analyze data:

Some tricky problems occurred when I used the software SPSS, which forced me to turn to other methods for help. To be frank, data analysis made me extremely anxious during that period of time.

Anxiety is a typical research emotion that the pre-service EFL teachers experienced, similar to Lee’s [35] findings. As with confusion, the participants’ anxiety in doing research can be largely ascribed to their lack of know-how. This negative emotion usually erupts during the middle stage of the research journey.

##### 4.1.1.3 Frustration

Frustration, the third negative emotion, was mentioned by six participants 17 times. According to their accounts, this feeling mainly arose because of two scenarios: the failure to successfully apply for a research project and literature reading. When the participants recollected their unsuccessful application experiences, they frequently touched upon frustration. For example, Missy admitted that the failure dealt a great blow to her:

After knowing that I did not manage to get the research project, I was actually frustrated. I had no idea how to deal with this programme since I had conceived of such a prototype. Should I forge ahead and finish it? At that time, I even considered giving it up and returning to my normal university life.

When it came to reading literature, frustration left a deep impression on Tyra.

Reading literature actually involves the process of looking for literature, but sometimes I have difficulty finding a paper on the basis of another. For example, some papers are covered in a monograph, which can only be accessed at a hefty price. Not being able to find the paper I want can be frustrating. This feeling bears some resemblance to the situation in which you are looking for clues, but the trail goes cold all of a sudden.

Frustration during the research process was mainly induced by external factors, from which the participants could draw some lessons. However, if frustration fails to garner due attention, pre-service teachers will likely become trapped in a vicious circle with other negative emotions, like self-doubt.

#### 4.1.2 Positive emotional experiences

Apart from negative emotional experiences, the participants reported nine positive emotions in their research. The three, which they mentioned the most often, are reported and analyzed as follows.

##### 4.1.2.1 Curiosity

Curiosity was the most intense positive emotion the participants mentioned in the interviews, which was always generated in the process of selecting a research topic. For the pre-service EFL teachers, doing research was uncharted territory and thus definitely held tremendous appeal. This feeling was the most conspicuous when they needed to decide upon a topic, because they were fascinated with almost every research area and had ambitions to make remarkable achievements. For Neal, curiosity became an incentive which stimulated him to become a teacher researcher:

For one thing, I was particularly curious about what research actually was. To be frank, I had no idea of it in the beginning, so it was curiosity that propelled me to know more about it. For another, I was also curious about whether I was capable of making some achievements in research. Just because I had not dabbled in this area before, I was eager to stretch myself and tap into my possibilities.

Curiosity experienced during the fledgling stage of doing research can be deemed an internal drive which holds the possibility of encouraging and motivating pre-service EFL teachers to confront potential challenges lying along the research journey.

##### 4.1.2.2 A sense of achievement

A sense of achievement was the participants’ second most frequently mentioned positive emotion, which was closely associated with their research productions, corroborating Borg’s [36] findings. For Olivia, this feeling was derived from her successful application for a research project, giving her the feeling that all of her great efforts had finally paid off:

When I knew that I succeeded in applying for this project, a sense of achievement welled up in my heart. Before this success, research seemed to be something I could never excel in. In other words, it was so far away from me. But this experience helped me increase my confidence and injected great momentum into my research work.

Some participants also reported a sense of achievement after reading a lengthy and complicated English paper, which bears some resemblance to venturing beyond a great mountain. The same applied for Missy:

In the process of reading literature, for instance, I often found the viewpoints in one paper inconsistent with those in another. After I absorbed and digested these different ideas and returned to my own study, I tried to perceive it from a novel perspective. This offered me a feeling that I was approaching what I intended to investigate, making me feel accomplished.

##### 4.1.2.3 Motivation

Motivation was usually generated at the same time as the sense of achievement. External acknowledgement and compliments were likely to function as the internal impetus motivating pre-service EFL teachers to forge ahead and continue their research work. For instance, Missy drew tremendous motivation from her successful application for a research programme:

The moment I knew I managed to get this research program and my design was evaluated as a key one, a sense of motivation overwhelmed me. It made me realize that my topic was of significantly practical value and I was driven to continue this study and make achievements.

Therefore, motivation can stimulate pre-service EFL teachers to increase their research competence. For instance, some may decide to seek further education. Therefore, more external support should be provided to trigger pre-service EFL teachers’ motivation to conduct research.

#### 4.1.3 Complex emotional experiences

The participants’ general impression of their research experience was that they were inundated with complex emotions, both positive and negative. The two most common experiences were pain and happiness, and love and hate.

##### 4.1.3.1 Pain and happiness

Pain and happiness represented a general feeling of the research work shared by the participants. First, this complex emotion was not confined to a particular event but permeated the whole process. Missy, for example, was imbued with passion and enthusiasm at the initial stage of her research journey, but she gradually lost her direction and confidence when she moved forward:

From my perspective, my research journey was filled with pain and happiness. What impressed me most was not only the excitement and passion in the early stage of doing research but also the happiness of acquiring knowledge and making progress in reading literature, especially when I found the answers to a question. But along this journey, I experienced confusion as well, unable to find the motivation and significance of doing research.

Second, this complex emotion could occur in connection with one specific event which seemed negative at first but turned out to be rewarding after they had worked hard. Olivia experienced this while writing a project proposal:

At this time, I needed to write a research plan, the process of which was filled with both pain and happiness. Actually, there was no definite research method for literary studies, but I had to write it in the proposal. I clearly knew that it was normal, so I tried to let myself gradually adapt to this process. After finishing submitting this proposal, I would like to describe this period of hard work as a honeymoon phase.

##### 4.1.3.2 Love and hate

Love and hate constituted another complex emotion that the participants reported as part of their research experience. It shared some similarities with pain and happiness but occurred at a deeper level. According to the participants, this emotion summarized their general feeling of the research experience. As Iris mentioned, doing research was the process of encountering problems and tackling them:

On one hand, I knew I had successfully applied for this project and should be responsible for carrying it forward. I had great passion and motivation to study what I was interested in. But on the other hand, I encountered insurmountable difficulties in this process as well, which would multiply with the development of the research. This was where I had a mixture of love and hate, a very complicated emotion.

The participants’ emotional stories conveyed why pre-service EFL teachers, as research novices, have more negative emotional experiences than others. Meanwhile, doing research also brought the participants some positive emotions, like curiosity and a sense of achievement. On the whole, pre-service EFL teachers’ emotional experiences in research can be described as complex.

### 4.2 Emotion regulation strategies used by pre-service EFL teachers in research

According to the data analysis, the emotion regulation strategies reported by the pre-service EFL teachers mainly fall into two categories – antecedent-focused and response-focused (see Table 5) – in line with Gross [27,28]. Generally speaking, the participants used response-focused strategies more often than antecedent-focused ones.

**Table 5:** Coding results of teachers’ emotion regulation strategies in research.

Emotion Regulation Strategies (142)		Coding Source	Coding Number	Percentage
Response-focused (90)	Communication	7	35	38.89
	Self-improvement	7	18	20.00
	Action taking	6	17	18.89
	Relaxation	7	10	11.11
	Adaptation	3	9	10.00
	Expressive suppression	1	1	1.11
Antecedent-focused (52)	Attention deployment	6	18	34.62
	Cognitive change	5	16	30.77
	Separation	5	7	13.46
	Expectation lowering	5	6	11.54
	Situation selection	4	5	9.62

#### 4.2.1 Response-focused strategies

Compared with antecedent-focused strategies, the participants employed response-focused strategies more often to regulate their felt emotions. These response-focused strategies were mainly communication, self-improvement, action taking, relaxation, adaptation and expressive suppression, with communication being the most favored one. In addition, the researchers identified a new type of response-focused strategy: self-improvement. Different from action taking, which was mainly utilized after positive emotions were evoked, self-improvement was associated with regulating negative emotions.

##### 4.2.1.1 Communication

Communication refers to the act of talking with supervisors, family members, other pre-service EFL teachers and so on in an attempt to find an outlet for negative emotions. When Tyra felt quite confused about reading literature, she was inclined to pour out the negative emotions to her friends and get some comfort:

When I had a feeling of confusion in reading literature, I would talk with my friends about why I failed to find the papers. It was through this method that I could regulate my emotions and continue doing my project, in which case I also unleashed negative emotions and became more preoccupied with my work.

In Emma’s case, she would communicate with others when she felt anxious and pressured in most cases. Furthermore, she perceived emotion regulation as a pivotal skill that pre-service EFL teachers should be equipped with:

I tend to actively adjust the feelings of anxiety and pressure. I do not pretend to be strong because holding back negative emotions would break me down. For me, emotion regulation is extremely crucial. If I do not regulate negative emotions, they would finally explode and affect my project.

This finding is similar to the finding in related foreign studies that teachers often adopt response-focused strategies, such as active communication and narrative inquiry [37,38], showing that communication is a significant emotion regulation strategy through which teachers manage to seek research support from others and vent their negative emotions.

##### 4.2.1.2 Self-improvement

Self-improvement refers to the act of taking steps to improve one’s personal research competence when one has difficulty with literature reading, data collection and data analysis. For instance, Neal took the initiative to practice his reading to enhance his ability to read papers more quickly and efficiently:

When I had negative emotions in literature reading, I would consciously carry out some reading training, such as memorizing more English words, reading more papers related to this topic and so on. I just told myself not to escape when encountering problems and tried to polish up my reading skills.

Self-improvement can not only enhance pre-service EFL teachers’ research capabilities but also help them regulate negative emotions. Consequently, they may become more motivated to do research. This finding is the same as Gu’s [26] finding that self-improvement can be deemed a requirement for teachers to pursue lifelong learning and promote professional development.

##### 4.2.1.3 Action taking

Action taking usually happened after the pre-service EFL teachers experienced positive emotions and felt motivated to conduct research. The emotional experiences the participants shared mainly focused on the intention of seeking further education to better themselves. For example, Olivia’s past research experiences inspired her to become a postgraduate student:

Later, it was also this period of research experience that made me think I was suitable for graduate school and that I was willing to devote a lot of time and energy to doing research. I even considered enrolling in a PhD programme to immerse myself in research. I was not saying that I had an excellent ability or talent, but I would rather be a teacher researcher.

This strategy adequately showcases the pre-service EFL teachers’ agency in doing research. They opted to move forward and make progress after receiving positive feedback from outside, which ought to be encouraged and advocated.

##### 4.2.1.4 Relaxation

The participants employed the strategy of relaxation mainly through doing exercise, listening to music, watching films and doing other activities to unwind. For example, Neal enjoyed doing some exercise to get a short respite from negative emotions:

At this time, I found that through the relaxation of doing exercise, I could completely get rid of the negative emotions I felt in paper writing and return to a relaxed physical and mental state. Through this strategy, I was able to continue my study in a good mood.

##### 4.2.1.5 Adaptation

Adaptation refers to the passive act of accommodating oneself to a particular situation or change. Sometimes, the pre-service EFL teachers were ill-equipped to tackle some unexpected problems, leaving them with no alternative but to adapt and accept these problems. Iris admitted that when she encountered a sudden change in the policy on pre-service EFL teachers’ undergraduate theses, she could not accept it:

When the policy came out, I thought it was totally unacceptable and I could not understand it at all. Later, I knew that if my thesis was not associated with linguistics, I might run the risk of not being able to graduate. Under the pressure, I had no alternative but to accept it.

It seems that when the pre-service EFL teachers could not contend with the research environment in the face of some unacceptable matters, they tended to adapt to the new context and try their best to accept the external conditions. Compared with action taking and self-improvement, this passive act is likely to result in some negative emotions. Therefore, apart from adaptation, pre-service EFL teachers must try to employ other strategies to regulate their research emotions.

##### 4.2.1.6 Expressive suppression

Expressive suppression refers to the act of restraining the expression of negative emotions. Surprisingly, only Missy reported employing this strategy in the interviews, which, to some degree, contradicts the findings of Taxer and Gross [9] and Gong et al. [8]:

Sometimes, I just bury these negative emotions in my heart – something to do with my personality – because I am worried about whether my negative feelings will affect the moods of my friends.

The participants tended to actively seek strategies to regulate their negative emotions instead of holding them back, thus displaying a new characteristic of pre-service EFL teachers in the new era. Among the participants, Neal is living proof:

Personally, I did not like negative emotions very much and I tried to reduce their detrimental impact on me. Instead, I would try amplifying the influence of positive emotions so that I would not be swayed by negative ones.

#### 4.2.2 Antecedent-focused strategies

The antecedent-focused strategies in this study mainly entailed attention deployment, cognitive change, separation, expectation lowering and situation selection, with attention deployment being the most frequently used strategy. Interestingly, the participants made no mention of situation modification in this study, which was the most effective one in Sutton’s [7] study.

##### 4.2.2.1 Attention deployment

Attention deployment in this study refers to the act of shifting one’s attention in the form of concentration and distraction, which concurs with Gross’s [28] findings. When an increasing number of problems occurred in the research process, Iris chose to calm herself down and focus on these difficulties:

When more and more problems arose, I usually calmed myself down first. Then I opened a Word document and made a list of all my questions one by one, trying to concentrate on the trouble and think carefully to find solutions.

This strategy demonstrates pre-service EFL teachers’ agency in the face of negative research emotions. Prior to the appearance of unpleasant emotions, the participants gave attention to the things that might bring them desirable emotions.

##### 4.2.2.2 Cognitive change

Compared with adaptation, the participants who used the strategy of cognitive change actively adjusted their perspective on certain things. First, they tended to perceive a situation through a positive lens. Iris is a case in point:

When I encountered some complicated problems in the process, I would reflect on my past research experience and recall my solutions. Then I comforted myself that, last time, I succeeded in addressing the difficulties and I would also overcome the obstacles this time. This gave me much relief.

Second, some participants compared their personal situation with that of a less fortunate person to find comfort, which was also mentioned in Gross’s [28] study. Regarding the research resources Olivia could access, she reevaluated what she had by comparison:

I really envied the research environment others could have, but I also told myself that at some universities, there were no literature courses at all. By contrast, I had access to British and American literature courses at our school, and some optional classes as well. It gave me the feeling that I fell short of the best but was better than the worst.

This echoes Jiang et al. [10], who concluded that cognitive change appears to be more effective than suppression. In the present study, the pre-service EFL teachers modulated the way in which they considered a problem and changed the nature of their emotions from negative to positive.

##### 4.2.2.3 Separation

Different from cognitive change, separation refers to the act of separating the emotions related to research from those related to one’s personal life [26]. It can be deemed a kind of life wisdom that pre-service EFL teachers make a clear differentiation between research and life and try to avoid some research emotions adversely impacting their daily lives. As Ossy mentioned in the narrative questionnaire, she tended to separate research from life to regulate her emotions:

When I faced hurdles, I liked to put aside the questions remaining to be tackled, and I enjoyed life for a while. After that, I returned to my research work and set about dealing with them.

##### 4.2.2.4 Expectation lowering

Negative emotions, to some extent, are the result of setting overly ambitious and even unrealistic goals. In this case, appropriately lowering the expectations for one’s research is likely to relieve anxiety and make the mind peaceful. According to Neal, lowering expectations was very helpful for him:

Sometimes I had extremely high expectations of myself, which filled me with anxiety. At this time, I consoled myself that I was just an undergraduate and pre-service English teacher. To be honest, the pressure of research was not that intense. For me, there was no such problem as the predicament of “publish or perish” facing professors. Therefore, I tried not to place a great strain on myself.

This finding indicates that different participants had different understandings of doing research. Sometimes, setting moderate but motivating goals is ultimately beneficial, especially for pre-service EFL teachers.

##### 4.2.2.5 Situation selection

Situation selection refers to the act of choosing a certain situation where pre-service EFL teachers would speculate that desirable emotions will arise. This strategy was less frequently employed by the participants but is effective because it plays a pivotal role in the first stage of emotion generation, with the likelihood of negative emotional experiences following. Tyra used this strategy for literature reading:

I had planned to read literature the night before, but when I woke up the next morning, I found myself not in the mood to do it. Then I would postpone reading papers. From my perspective, if I kept storing up negative emotions in the process of doing research, I would hate it even more.

This supports the study done by Uzuntiryaki-Kondakçı et al. [31]. Situation selection reflects teachers’ conscious selection of a specific context that is likely to be pleasant.

### 4.3 Influencing factors of pre-service EFL teachers’ emotion regulation in research

As shown in the coding results (see Table 6), two factors were at play in the pre-service EFL teachers’ emotion regulation in conducting research: personal and environmental factors. They both played significant roles in influencing the participants’ choice of different regulation strategies, with each being mentioned 21 and 19 times respectively.

**Table 6:** Coding results of influencing factors of teachers’ emotion regulation in research.

Influencing Factors (40)		Coding Source	Coding Number	Percentage
Personal (21)	Past experiences	6	10	47.62
	Agency	6	6	28.57
	Personality	4	5	23.81
Environmental (19)	Relationships with others	7	10	52.63
	Research support	7	9	47.37

#### 4.3.1 Personal factors

Personal factors appear to exert a greater influence on emotion regulation than environmental ones, which mainly include past experiences, agency, and personality. Past experiences were mentioned by the participants the most (10 times), followed by agency (six times) and personality (five times).

##### 4.3.1.1 Past experiences

Past experiences in this study are not confined to previous research experiences but cover teaching practicum experiences as well. Past research experiences not only enabled the participants to accumulate valuable lessons on how to do research but also to have a good command of how to appropriately regulate different research emotions. Iris cherished her research experiences very much:

If I had not participated in this project, I may not have accumulated so much valuable experience. Be they positive emotional experiences or negative ones, I managed to adjust my emotions again and again and finally broke loose from them. In this process, I also made huge strides in retrieving and managing literature, writing papers and communicating with others.

Apart from past research experiences, practicum experiences emerged as conducive to pre-service EFL teachers’ emotion regulation. Take Ossy for example. Her practicum experience taught her an effective method for dealing with personal pressure:

The emotional impact of this practicum experience appeared to be my improved stress tolerance, which enhanced my personal confidence to a great degree when it came to my undergraduate thesis. I would not become anxious easily and tended to adopt a more peaceful state of mind.

This strategy aligns with the discovery made by Hosotani and Imai-Matsumura [32] that past experiences contribute to more effective emotion regulation, in which case teachers actively associate what they experienced with what they are experiencing in order to reduce the negative impact of research emotions to the greatest extent possible.

##### 4.3.1.2 Agency

Agency can be interpreted as an incentive for pre-service EFL teachers’ conscious and active attempts to modify a less favorable situation in their research [39]. Consequently, they may manage to down-regulate negative emotions and maintain better mental health. Neal shared his strategy for coping when meeting obstacles in research:

Firstly, I was willing to do research. Therefore, in the face of some hurdles, I would take the initiative in choosing some measures to regulate my research emotions instead of allowing myself to be swayed by them. From my perspective, my personal agency contributed a lot to my positive emotional experiences in research.

As mentioned in Gu’s [26] qualitative study, agency functions as a facilitator, which prompts pre-service EFL teachers to actively seek strategies to adapt to or change the environment. Consistent with the definition of teacher emotion, this appropriately showcases the participants’ transformative power in the research context, which deserves to be encouraged and advocated.

##### 4.3.1.3 Personality

Personality, to some extent, can also exert an influence on pre-service EFL teachers’ emotion regulation, both in beneficial and detrimental ways. Some pre-service EFL teachers, especially the extroverts were loath to bury negative emotions. Neal is one of them:

It has something to do with my personality. Actually, I do not like to hold back these negative emotions. Some of my classmates quite enjoy the feeling of anxiety brought by exams and they deem it a strong motivation. However, I am inclined to down-regulate negative emotions and up-regulate positive ones.

Comparatively introverted pre-service EFL teachers, however, preferred suppressing felt emotions to expressing them. Pouring out negative emotions to others, in the teachers’ view, becomes an emotional burden for themselves. Missy identified strongly with this point:

Sometimes, I tend to bury these negative emotions in my heart – something to do with my personality – because I am worried about whether my negative feelings will affect my friends’ moods.

There is no distinction between a good or bad personality, but personality indeed plays a fundamental role in the process of emotion regulation. However, this influencing factor seems to have received little mention in previous studies and therefore merits further exploration.

#### 4.3.2 Environmental factors

Environmental factors also contribute significantly to pre-service EFL teachers’ emotion regulation. In this study, this type of factor mainly covers pre-service EFL teachers’ relationships with others (e.g. supervisors, family members, other pre-service EFL teachers) and the research support available to them (e.g. research policy, research funds). Inconsistent with the findings of Yin and Lee [40], the participants did not mention expressive rules.

##### 4.3.2.1 Relationships with others

Pre-service EFL teachers’ relationships with their supervisors, family members, friends and other pre-service EFL teachers determine their options for strategies to a certain extent. When the participants encountered research problems, they first turned to their supervisors for help, from whom they could receive immediate and useful instruction. In this case, relationships with supervisors were described as a kind of positive interaction. The same goes for Neal:

If I have established a close and intimate relationship with this teacher, I will definitely give priority to seeking his help the moment I encounter difficulties and some negative emotions. On the contrary, if the teacher does not leave the impression of being easygoing on me, then next time I may not choose to consult him when I am in a bad mood.

This finding indicates that if intimate relationships exist between the participants and others, chances are that they would interact with them in an effort to disclose their personal emotions, with the strategy of communication complementing this act. As indicated in Chang’s [29] case study, this factor is ultimately beneficial to teachers’ better emotion regulation.

##### 4.3.2.2 Research support

Research support can function as both a boost and hindrance to pre-service EFL teachers. They are constantly interacting with the complex research environment, where they deploy their personal agency to regulate different emotions. On one hand, the research support provided by the university can trigger enthusiasm and motivation. For instance, Neal shared his opinions on the research incentives he received:

From my perspective, we as undergraduates do not have many opportunities to do research. In this case, the research projects organized by my school actually offer me great motivation to learn and conduct research, which plays the role of a mediator to facilitate my emotion regulation. What’s more, the incentives such as scholarships and funds all imbue me with positive emotions. For example, the research funding I received gave me a sense of achievement, which turned out to be not only financial aid but also outside recognition for my research work.

On the other hand, insufficient research support may accelerate the generation of negative emotions, having an indirect effect on emotion regulation. As Tyra shared, limited access to academic lectures exacerbated her negative emotions, impelling her to improve her personal skills.

This finding sheds light on the significance of increasing research support for pre-service EFL teachers so they may feel more motivated to conduct research. Some participants mentioned that there was still room for the university to make improvements. For example, Iris talked about the possibility of offering courses on emotion regulation to pre-service EFL teachers:

I think this kind of course is quite necessary because whether in research or in the competitive environment we are now facing, many people are under intense pressure. If we fail to find an outlet to unwind, we may suffer from an irreversible impact of negative emotions.

## 5 Discussion

### 5.1 The complex features of emotional experiences in research

The participants’ emotional experiences in conducting research can be described as complex, with positive and negative emotional stories intertwined. This, to a certain extent, is consistent with the aforementioned research findings [[24], [25], [26]] showcasing that doing research is a practice for pre-service EFL teachers which involves emotional labor.

Meanwhile, the coding results indicate that negative emotions in doing research were mentioned more frequently by the participants compared with positive and complex ones, and they were especially felt during the fledgling and progressing stages of research, when the participants were encountered with some problems with topic selection, literature reading, data collection and data analysis. This does not align with Hargreaves’ [41] finding that teachers at the beginning stage of doing research were more likely to feel enthusiastic and optimistic. This contradiction can be attributed to the fact that pre-service EFL teachers are lacking in research learning and experience as novices, which inevitably causes them to be imbued with negative feelings during the initial stage. In addition, the emotional experiences reported by the participants were interwoven, which means that the pre-service EFL teachers experienced both positive and negative emotions throughout the research journey. This gives researchers insight into the complex feature of teacher emotion in doing research, which is involved in a dynamic system.

### 5.2 The diverse types of emotion regulation strategies in research

The emotion regulation strategies in conducting research that the participants described mainly fall into two categories: antecedent-focused and response-focused. This supports the framework introduced by Gross [27,28]. Furthermore, the participants employed response-focused strategies more often than antecedent-focused ones, which showcases that pre-service EFL teachers tend to regulate their emotions after, instead of before, their occurrence.

Apart from the emotion regulation strategies mentioned in Gross’s [27,28] framework, this study has found some new ones, such as communication, self-improvement and action taking. This, to a certain extent, enriches the existing studies on teachers’ emotion regulation strategies. Take communication for example. Different from suppression, as was discovered by Gong et al. [8] and Taxer and Gross [9], communication is the active act of talking with others in an attempt to find an outlet for negative emotions, which demonstrates the agency of pre-service EFL teachers to pursue their personal development and adapt to the complex and dynamic environment. Regarding the strategy of action taking, it usually happens after pre-service EFL teachers have positive emotions and feel motivated to conduct research. This is consistent with Gross’s [27] assumption that emotion regulation involves the modulation of both negative and positive emotions. In conclusion, the findings exhibit the diversity of emotion regulation strategies used by the pre-service EFL teachers in this study.

### 5.3 The interplay between influencing factors of emotion regulation in research

The influencing factors of emotion regulation in doing research that the participants described mainly comprise two types – personal and environmental – in accordance with the aforementioned research [3,26,29,32]. Personal and environmental factors interact, which demonstrates that teacher emotion lies in its interaction and transaction with the environment [20].

Regarding environmental factors, the participants frequently mentioned the positive role they played in their emotion regulation processes. This is inconsistent with what was uncovered by Gu [26], who depicted the negative interplay between teacher emotion regulation and the environment. This indicates the significant role that environmental factors play in teacher emotion regulation and highlights the necessity for policymakers to develop more favorable research policies and organize more inspiring research activities.

## 6 Conclusions, implications, and limitations

The major conclusions of the study can be summarized as follows. First, the participants experienced positive, negative and complex emotions associated with conducting research, among which the negative ones left the deepest impression on them, with confusion being the most frequently mentioned one. Doing research also brought them some positive emotions, like curiosity and a sense of achievement. On the whole, pre-service EFL teachers’ emotional experiences in conducting research can be described as complicated.

Second, the participants employed both antecedent-focused and response-focused strategies to regulate their research emotions, with response-focused ones being more favored by them. Communication was their most frequently used strategy, showcasing the participants’ active search for external help with their emotion regulation. Expressive suppression was rarely mentioned in this study, denoting the tendency to express felt emotions instead of suppressing them.

Third, both personal and environmental factors played roles in the pre-service EFL teachers’ emotion regulation in doing research. From the personal aspect, past experiences, agency and personality contributed to the participants’ different reactions to emotional experiences. From the environmental aspect, their relationships with others and the research support they received exerted a great influence on how they regulated their emotions.

In light of these findings, some implications can be derived for pre-service EFL teachers, educators, and policymakers. First, as research novices, pre-service EFL teachers lack systematic learning of how to do research, which may result in negative emotional experiences. Worse still, they receive little training in how to perceive and regulate research emotions appropriately. Considering the indispensable role of research emotions in teachers’ well-being and research, there is an urgent need for pre-service EFL teachers to receive formal training not only in research but also in emotion regulation. Second, educators should ramp up communication and interaction with pre-service EFL teachers to provide them with academic and mental instruction, as some participants mentioned that specialized courses on emotion regulation would offer them an opportunity to have emotional exchanges with others and unleash especially negative emotions. Third, policymakers must take the features of teacher research and teacher emotion into consideration, especially those of pre-service EFL teachers. Gaining a better understanding of pre-service EFL teachers’ possible emotional experiences and emotion regulation strategies will enable policymakers to develop more favorable research policies and more inspiring research activities to improve the research environment and atmosphere.

Due to the complexity of the interaction between pre-service EFL teachers and the research environment, there are two main limitations to this study. First, the sample size of seven pre-service EFL teachers is small. With the qualitative data analyzed, the interpretations of the participants’ emotional stories about conducting research appear descriptive and thus fail to conjure a comprehensive picture of all pre-service EFL teachers’ emotional experiences. Future studies should have larger research samples and take account of the research backgrounds of different participants to explore more diversified and profound emotional stories. Second, considering the dynamic nature of teacher emotion, it would be impossible for the participants to have a complete recall of their emotional experiences in conducting research without any omission. In addition, their recollection appeared somewhat subjective. All these factors may have a bearing on the reliability and validity of the research data. Future studies can employ other measures to capture pre-service EFL teachers’ emotion regulation and combine qualitative and quantitative methods.

## Ethics approval and consent to participate

All methods were carried out in accordance with the guidelines and regulations of the 1964 Helsinki declaration and its later amendments. Since this study does not involve intervention and it is low risk, ethical review and approval were waived according to the institutional review boards at School of Foreign Language in Soochow University.

Each participant was required to sign a written form of informed consent. All participants were informed about the aim of the study and their right to withdraw from the study at any time. All methods were carried out in accordance with relevant guidelines and regulations.

## Consent for publication

Not applicable.

## Availability of data and materials

Data will be made available on request.

## CRediT authorship contribution statement

**Renquan Heng:** Writing – review & editing, Writing – original draft, Methodology, Investigation, Data curation, Conceptualization. **Qi Cheng:** Writing – review & editing, Writing – original draft, Formal analysis, Conceptualization. **Liping Pu:** Writing – review & editing, Writing – original draft, Methodology, Formal analysis, Conceptualization. **Qingyu Tang:** Writing – review & editing, Writing – original draft, Methodology, Data curation.

## Declaration of competing interest

The authors declare that they have no known competing financial interests or personal relationships that could have appeared to influence the work reported in this paper.

## Figures and Tables

**Table:** 

Number	Question	Prompts
Question 1	The common positive research emotions include curiosity, happiness, satisfaction, love, passion, pride, a sense of achievement and others; the common negative research emotions include confusion, frustration, fear, anxiety, helplessness, pressure, boredom, and others; the common complex research emotions include love and hate, pain and happiness, and others. Please browse and select some of the feelings you experienced during your research experiences. You are welcome to add new emotional words that are not included in the list.	1) Which emotion impressed you the most? 2) What happened? Could you give a detailed explanation for it?
Question 2	I have got several pictures related to your research experiences. Please recall what happened in that episode.	1) What happened at that time? 2) What emotional experiences did you have?
Question 3	You selected several words related to positive emotions from the list (if any). Can you make a detailed sharing about how you handled or regulated these positive emotions?	1) Why did you have this kind of emotions? What happened at that time? 2) What did you do in the face of them?
Question 4	You selected several words related to negative emotions from the list (if any). Can you make a detailed sharing about how you handled or regulated these negative emotions?	1) Why did you have this kind of emotions? What happened at that time? 2) What did you do in the face of them?
Question 5	You selected several words related to complex emotions from the list (if any). Can you make a detailed sharing about how you handled or regulated these complex emotions?	1) Why did you have this kind of emotions? What happened at that time? 2) What did you do in the face of them?

## Appendix I Narrative questionnaire

1) So far, I have done research for______(time). The development of my project is______. In the process of doing research, my attitude towards it can be summarized as______. From my perspective, doing research is______.
2) The school’s research requirements for pre-service EFL teachers are ______. The school will reward or punish pre-service EFL teachers for their research performance, such as______. In terms of these practices, I feel______, because______.
3) My research interest has experienced the change of______, because______. I _______ read literature (frequency). I read literature for______, and at the same time I find reading literature______. I often discuss research questions with______ because______. In addition, I______ (frequency) participate in the research activities organized by the university or school, such as______. I find these activities______.
4) I think the most difficult thing about doing research lies in______, this is because______. When I meet difficulties, I often deal with them in this way: ______.
